# Semicircular Canals Circumvent Brownian Motion Overload of Mechanoreceptor Hair Cells

**DOI:** 10.1371/journal.pone.0159427

**Published:** 2016-07-22

**Authors:** Mees Muller, Kier Heeck, Coen P. H. Elemans

**Affiliations:** 1 Experimental Zoology Group, Wageningen University, 6709 PG Wageningen, The Netherlands; 2 Leiden University, Dept. of Physics, Niels Bohrweg 2, 2333 CA Leiden, The Netherlands; 3 Sound Communication Group, University of Southern Denmark, 5230 Odense M, Denmark; Universität Bielefeld, GERMANY

## Abstract

Vertebrate semicircular canals (SCC) first appeared in the vertebrates (i.e. ancestral fish) over 600 million years ago. In SCC the principal mechanoreceptors are hair cells, which as compared to cochlear hair cells are distinctly longer (70 vs. 7 μm), 10 times more compliant to bending (44 vs. 500 nN/m), and have a 100-fold higher tip displacement threshold (< 10 μm vs. <400 nm). We have developed biomechanical models of vertebrate hair cells where the bundle is approximated as a stiff, cylindrical elastic rod subject to friction and thermal agitation. Our models suggest that the above differences aid SCC hair cells in circumventing the masking effects of Brownian motion noise of about 70 nm, and thereby permit transduction of very low frequency (<10 Hz) signals. We observe that very low frequency mechanoreception requires increased stimulus amplitude, and argue that this is adaptive to circumvent Brownian motion overload at the hair bundles. We suggest that the selective advantage of detecting such low frequency stimuli may have favoured the evolution of large guiding structures such as semicircular canals and otoliths to overcome Brownian Motion noise at the level of the mechanoreceptors of the SCC.

## Introduction

**Fig 1:**
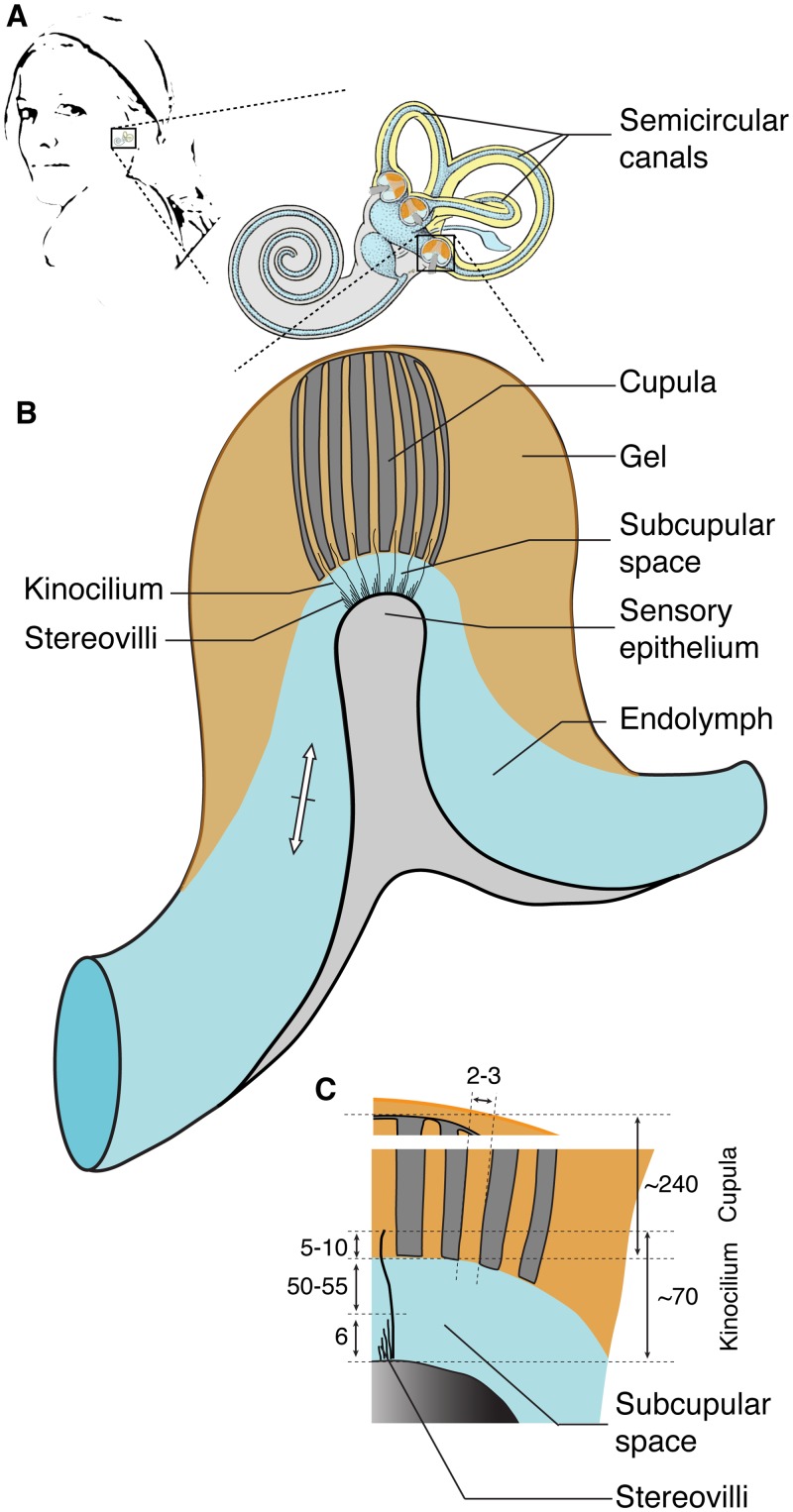
Schematic overview of location and dimensions of the mechano-electrical transducer system in a generalised vertebrate semicircular canal system. (**a**) *In situ* position and general shape of the vertebrate labyrinth with the semicircular canals (modified after [13]). (**b**) Schematic overview of a single sensory ampulla. The semicircular canal is filled with endolymph fluid (light blue) that is displaced during head rotation (white arrow). The cupula (dark grey) is connected to the roof of the ampulla and embedded in a mass of mucopolysaccharide gel (orange). The sensory epithelium (light grey) contains hair cells with apical hair bundles consisting of stereovilli and one central kinocilium. The fluid flow of ampullar endolymph at the sensory epithelium is limited to the subcupular space between the sensory epithelium and cupula. (**c**) Schematic overview and dimensions of the cupula and apical hair bundles. The kinocilia tips penetrate tubuli in the cupula and can move freely radially and slide longitudinally, allowing Brownian Movement of the hair bundles. Dimensions are indicated in μm.

The vertebrate semicircular canal (SCC) system helps coordinate body movement, including stabilization of an animal’s visual gaze during locomotion [1]. Specifically, this sensory system measures head rotation and consists of mutually connected toroidal loops filled with endolymph fluid. This fluid is displaced in response even to very low-frequency (0.01–10 Hz) angular movement [2–4], leading to highly viscous flow (Reynolds number 0.5 [2]). The SCC endolymph displacement deflects apical hair bundles of hair cells (Fig 1) causing sensory transduction through the gating of mechanosensitive ion channels [5,6]. A large morphological diversity of hair cell bundle morphology, e.g. kinocilia and stereocilia dimensions and arrangements is present throughout the metazoa ([7] and references therein). Hair bundles are subject to Brownian Motion or thermal noise [8–10], that results from the thermal agitation of water molecules, as first observed by Brown [11] and explained by Einstein [12] for freely diffusing particles. For cochlear and saccular hair cells, thermal (Brownian) noise amplitude varies inversely with frequency [8,9], which impedes the detection of low frequencies due to a decreasing signal-to-noise ratio.

However, it is not well known how Brownian Motion noise affects the detection of hair bundle movements in SCC. In the cochlea, the effects of Brownian motion on a hair bundle displacement [14–16] are in the order of 1 nm. Free ampullary hairs of the glass eel (*Anguilla* sp.) demonstrate a 68 nm root-mean-square (rms) displacement due to Brownian Motion [17], a value that is almost two orders of magnitude higher than those observed in cochlear hair bundles and therefore surprisingly large [14,17]. Micromechanical models [10] and measurements [17] show that hair bundle displacement due to thermal noise increases at the low frequency end of a cochlear hair cell’s frequency sensitivity range. Because head movements provide very-low frequency input, i.e. for humans the time constants are 5 ms and 20 s, with a natural frequency of 0.5 Hz (an elaborate survey of these quantities can be found in [2]), the influence of thermal noise due to Brownian motion at the hair bundles of the SCC can be expected to be even stronger. The detection of very-low frequency head movement may thus be impeded due to a decreasing signal-to-noise ratio.

What constitutes the actual micro-mechanical stimulus to SCC hair bundles is debatable. The apex of each hair cell contains hair bundles of which the single slim kinocilium is about 70 μm long [18]. In eel, about 10 μm of the kinocilia tips (1/6 of the kinocilium height) is embedded in 2–3 μm wide, mucopolysaccharide-filled channels in the gelatinous cupula [19] (Fig 1), which is anchored onto the roof of the ampulla. The cupula is generally thought to provide the mechanical stimulus to the hair cells based on experiments where cupula removal led to reduced activity in the afferent nerve [18,20]. However, experiments by Suzuki and colleagues [20] in frogs support an alternative hypothesis. They repositioned the cupula after initial removal, and observed that the neural response fully recovered due to repositioning [20]. The cupula now must have pressed on the kinocilia tips, as it is highly improbable that all kinocilia tips moved back into their canals and reconnected to the cupula. In addition, they also repositioned the cupula upside-down which also recovered the neural response [20], albeit partial.

These experiments provide support for the hypothesis that it is not the cupula but the viscous endolymph flow in the subcupular space that provides the actual stimulus to the hair bundles [3,21,22]. Contrary to the current view that the cupula would move the hair bundles, these data suggest that the endolymph presses on the hair bundles and deflects them. In turn, the hair bundles move the base of the cupula. Additionally, the kinocilia do not break upon cupula removal, which strongly suggest they are not firmly anchored into the cupula [18,20,23] and potentially even slide inside its tubules (Fig 1). It is therefore highly probable that the cupula acts as a stabilizing, guiding and protecting structure for the hair bundles and the endolymph flow, instead of a structure which mechanically mediates the hydrodynamic force of the endolymph to the hair bundles as suggested earlier [21]. Supporting this view is the fact that a cupula is not present in the SCC of hagfish [24]. The presence of the cupula may be crucial for SSC functioning, however we argue it is so not because it directly moves the kinocilia, but because it has strong effects on the subcupular fluid flow of the endolymph that provides the mechanical stimulus to the kinocilia [3].

In rest, the tips of the hair bundles were observed to show Brownian Motion with a magnitude of ca. 70 nm [17]. This Brownian Motion is perpendicular to their long axis. The cupular tubuli are wide enough (2–3 μm) to allow for this motion (although the observation of 70 nm was done for detached bundles). As far as we know, no information is currently available regarding Brownian movement of the hair bundles when they are stimulated by the endolymph flow when the head is rotated and/or when the kinocilia slide longitudinally in the tubuli of the cupula.

Here, we test the hypothesis that SCC hair cells have adapted hair bundle properties to circumvent signal input masking by Brownian Motion. To test this hypothesis, we performed numerical experiments using biophysical models of hair bundles. We found strong agreement between our model’s predictions and pre-existing experimental observations. Brownian Movement consists of white noise that is low-pass filtered by the water molecules and by the biological structures we are interested in. Our models suggest that at frequencies <10 Hz hair bundle properties require changes in material properties and morphology to circumvent Brownian Motion masking. These adaptations would allow for the successful detection of very low frequency mechanical stimuli. Consequently, we observe that SCC hair cells require about a hundredfold larger input stimuli compared to e.g. cochlear cells, and we argue this is to circumvent Brownian motion overload at the hair bundles. Furthermore, we suggest that the selective advantage of detecting such low frequency stimuli has shaped the evolution of large guiding structures, such as the semicircular ducts, to circumvent the masking effects of Brownian motion noise at the mechanoreceptor hair bundles.

## Methods

### Hair bundle model

We performed numerical experiments using biophysical models of hair bundles. To consider the excitation of hair bundles by physiological stimuli, the system can be considered a damped mass-spring system that ranges from under-damped, with a damping ratio <1, such as lateral line and auditory cells, to heavily over-damped, with a damping ratios of about 30 such as ampullar cells, as described in detail in e.g. [2,3].

**Fig 2:**
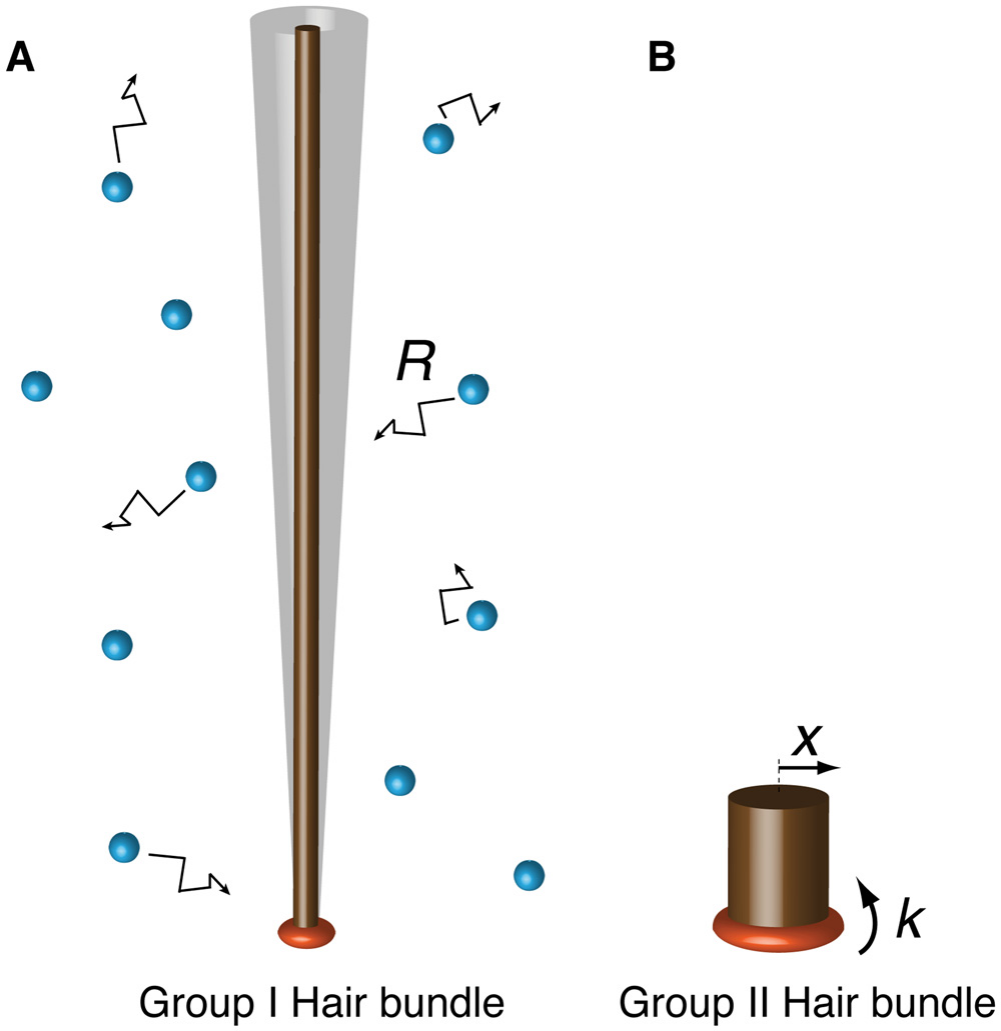
Schematic representation of hair bundle models. **a**, low-frequency Group I hair bundle and **b**, the high-frequency Group II hair bundle. Groups of water molecules (blue spheres) bombard a Group I hair bundle, which results in Brownian motion (BM) of the hair bundle tip. The cumulative bombardments result in a net force (*R*). Both hair bundles exhibit displacement (*x*) around a mean and are elastically coupled to the base with elasticity constant *k*.

To consider excitation of the hair bundles by Brownian Motion, a different approach is required. We model hair bundles as stiff rods that are elastically connected to the cell body at their base [25]. The bundle is bombarded sideways (i.e. in two dimensions) by groups of water molecules, which evoke the Brownian motion (Fig 2). Concurrently frictional and elastic forces counteract the Brownian Motion-force. To allow for the huge morphological and structural diversity of hair cells, their kinicilia and stereovilli arrangements (see e.g. [7]) we consider a rod as an elongated body with an equivalent radius to which Stokes’ law for viscous flow is applicable. The tip of the hair bundle moves randomly about its mean position and has a characteristic rms excursion.

We aim at providing a thorough analysis of Brownian motion mechanics to clarify our assumptions and ensure correct equation dimensionality. We assume linear elasticity at the hair bundle base as outlined in [25] and applying Hooke’s law. A force-balance equation as a function of time provides the equation of motion (see Table 1 for symbols, quantities and used parameter values):

$$m\cdot \frac{d\dot{x}\left(t\right)}{dt}=R\left(t\right)-\gamma \cdot \dot{x}\left(t\right)-\kappa \cdot x\left(t\right)\;[\text{N}]$$ (1)

with *x(t) =* hair bundle tip displacement, *R*(*t*) = the Brownian Motion force, *γ* = the friction coefficient, *γ = 6π⋅η⋅r⋅r*_*eq*_ (Stoke’s law for a prolate rod with radius *r* and a correction factor for cylindrical bodies *r*_*eq*_), and κ = the elasticity constant of the hair bundle. We could not find any information about distributed Brownian Motion bombardment of a cylindrical rod. We thus modelled the hair bundle as an elastically bound particle with the mass of the entire bundle and a dimensionless spring to determine the inertial and elastic forces, and as a stiff cylindrical rod to determine the friction-forces. This hair bundle is hit by water molecule clusters causing the Brownian Movement. Inertial forces are negligible as argued below. We also considered complexities due to rotation of the hair bundle to be of minor importance, as the hair bundle-excursions are small. The resulting model is a stiff, cylindrical elastic rod with mass *m* and subject to friction and thermal agitation.

**Table 1:** Symbols and values of used quantities.

Symbol	Physical quantity	Group I (r_eq_ = 10)	Group II (r_eq_ = 1)	units
*A*,*a*,*B*,*b*	local constants (Eqs 8–10)			
*D*	diffusion constant: *D = kT/γ*	3.8E-14	6.4E-14	m^2^/s
*f*	frequency			Hz
*f_c_*	corner frequency *f*_*c*_ *= λ/T*_*c*_			Hz
K	the Boltzmann constant	1.38E-23	1.38E-23	J/°K
M	mass of hair bundle	1.13E-13	5.24E-15	kg
l	length of hair bundle	65E-6	7E-6	m
*r*	radius of hair bundle	0.5E-6	3E-6	m
*r*_*eq*_	equivalent radius	10	1	
*s*	Laplace-operator			Hz
*R(t)*	Brownian noise force			N
t	time			s
*T*_*0*_	absolute temperature	293	293	°K
*T*_*c*_	time constant: *T*_*c*_ *= γ/k*	2.4	1.3E-4	s
*x*, $\dot{x}$	hair bundle displacement (with derivative)			m
X*(0)	Brownian motion hair bundle-tip (rms)	68E-9 [17]	0.6E-9	m
γ	friction coefficient:*γ = 6π·η·r⋅r*_*eq*_	1.06E-7	6.35E-8	kg/s
*η*	dynamic viscosity	1.124E-3	1.124E-3	Pa·s
κ	Elasticity constant	4.4E-8	500E-9 [5,9]	N/m
*λ*	*x*^*2*^			m^2^
Λ	Laplace transform of *λ*			m^2^/Hz
ω	angular frequency			rad/s
τ	time constant	1.2	0.065E-4	s

The values with an equivalent radius of *r*_eq_ = 1 are represent those of a freely moving (i.e. without elasticity) spherical particle with a radius of 3 μm. The column for *r*_eq_ = 10 represents values relevant for a freely moving prolate rod with a length/radius ratio of 30–60 and a radius of 0.5 μm.

Because we are interested in the frequency-spectrum of the sensitivity, we transform (Eq 1) into the frequency domain using the Laplace-transform. This cannot be done directly as the Laplace-transform of the noise force *R*(*t*) is unknown to our knowledge. Therefore, we modify (Eq 1) to an energy-balance equation by multiplying it by *x*, applying Boltzmann’s equipartition theorem in two dimensions (because of the sideward’s bombardment of the rod) and introducing *λ = x*^*2*^, which yields:

$$m\ddot{\lambda }+\;\gamma \dot{\lambda }+2\kappa \lambda =4kT\;\left[\text{J}\right]$$ (2)

(Eq 2) is the basic equation describing a balance of Brownian Motion energies, including thermal agitation of water-molecules, inertia and elasticity of the hair bundle, and hydrodynamic friction. The derivation of Eqs (1) to (2) can be found in detail in [25], section 15.6 and is not repeated here. As can be seen in (Eq 2), frictional- and elastic forces counteract the Brownian Motion-force on the hair bundle. However, as argued by Einstein [26] (section 2) for freely diffusing particles, the influence of inertial forces can be ignored because of the relative large mass of these particles compared to the bombarding water molecule clusters. For a hair bundle inertial forces can thus also be ignored because its relative mass is much larger than the mass of a freely diffusing particle. This approach also justifies that we consider tip-excursions of the hair bundle rather than the excursion of its centre of mass.

These considerations reduce (Eq 2) to:

$$\;\gamma \dot{\lambda }+2\kappa \lambda =4kT\;[\text{J}]$$ (3)

To simplify (Eq 3) we introduce the diffusion constant (*D = kT/γ*) and the characteristic time constants for energy (τ) and displacement (*T*_c_) respectively (*τ = γ/2k = T*_*c*_*/2*), which yields:

$$\tau \dot{\lambda }+\lambda =4D\tau \;[{\text{m}}^{2}]$$ (4)

To solve and obtain *λ* we perform the Laplace transform of (Eq 4):

$$\Lambda \left(s\right)=\left(4D\tau \right)\left(\frac{1}{s}\right)\left(\frac{1}{s\cdot \tau +1}\right)\;[{\text{m}}^{2}/\text{Hz}]$$ (5)

and back-transform (Eq 5) to the time-domain:

$$\lambda =\left(4D\tau \right)\left(1-e^{-\frac{t}{\tau }}\right)\;\left[{\text{m}}^{2}\right]$$ (6)

(Eq 6) describes the development of the power (*λ*) starting from a hypothetical rest-situation at *t* = 0 under influence of bombardments of water molecule groups. Since $x=\sqrt{\lambda }$, the development of the average displacement (*x*) to a final rms value is:

$$x=\sqrt{4D\tau }\sqrt{\left(1-e^{-\frac{t}{\tau }}\right)}\;[\text{m}]$$ (7)

As we are interested in hair bundle response in the frequency domain, we need to perform the Laplace-transform again. However, (Eq 7) is difficult to transform directly and therefore we chose an alternative approximation for (Eq 7) as the sum of two exponential functions, which is easy to transform:

$$x=\sqrt{4D\tau }\cdot \left[A\left(1-e^{-\frac{t}{a\tau }}\right)+B\left(1-e^{-\frac{t}{b\tau }}\right)\right]\;[\text{m}]$$ (8)

in which A+B = 1. The values A = 0.25, a = 0.033, B = 0.75 and b = 0.78 result in a small error of 3% between Eqs (7) and (8). To avoid unrealistic large errors for small values of *t*, the error is considered with respect to the maximal values of Eqs (7) and (8). The Laplace-transform of (Eq 8) is:

$$X\left(s\right)=\sqrt{4D\tau }\cdot \left(\frac{1}{s}\right)\left[A\left(\frac{1}{s\cdot a\cdot \tau +1}\right)+B\left(\frac{1}{s\cdot b\cdot \tau +1}\right)\right]\;\left[\text{m}/\text{Hz}\right]$$ (9)

The frequency-dependent behaviour of the hair bundle is obtained by letting *s → jω* and determining the Bode-amplitude-spectrum:

$$X^{*}\left(f\right)=\;f\cdot X\left(f\right)=\frac{1}{\pi \sqrt{2}}\cdot \sqrt{\frac{kT}{\kappa }}\left[A\left(\frac{1}{\sqrt{\pi^{2}f^{2}a^{2}T_{c}{}^{2}+1}}\right)+B\left(\frac{1}{\sqrt{\pi^{2}f^{2}b^{2}T_{c}{}^{2}+1}}\right)\right]\;[\text{m}]$$ (10)

(Eq 10) described the spectrum visualized in Fig 2. As *T*_*c*_ *= γ/κ*, this quantity depends on elasticity, friction and bundle radius.

The low-frequency plateau of (Eq 10) is:

$$X^{*}\left(0\right)=\;\frac{1}{\pi \sqrt{2}}\cdot \sqrt{\frac{kT}{\kappa }}\;[\text{m}]$$ (11)

which is independent of *γ* and therefore of both friction and bundle-radius. The used parameter values are listed in Table 2.

**Table 2:** Construction of hair cell amplitude spectra.

Line#	Organ	Cells	Genus	Source	Roll-off (Hz)	Max displacement (nm)	Transfer
1	Otolith	Utricular	*Chinchilla*, *Homo*	Model [27]	1.6 LP	3.5e3	x/y”
2	Otolith	Utricular	*Saimiri*, *Homo*	Model [28]	0.032 BP 9.95	3.5e3	x/y”
3	Statocyst	C1-crista	*Octopus*, *Allotheutis*	Measurement [29,30]	0.25 BP 1	5e3	x/y”
4	Statocyst	C2-crista	*Octopus*, *Allotheutis*	Measurement [29,30]	0.1 BP 1	5e3	x/y”
5	Ampulla	HC-crista	*Opsanus*	Measurement [31]	0.03 BP 15	10e3	x/y’
6	Ampulla	All cristae	*Homo*	Model [2]	0.008 BP 31.8	10e3	x/y’
7	Cochlea	3^rd^ turn outer hair cells (OHC)	*Cavia*	Measurement [32]	220 HP	400	x/y
8	Cochlea	4^th^ turn OHC	*Cavia*	Measurement [32]	700 HP	400	x/y
9	Cochlea	4^th^ turn OHC	*Cavia*	Model [32]	700 HP	400	x/y
10	Lateral line	Supraorbital	*Acerina*	Measurement [33]	118 HP	115	x/y
11	Lateral line	Supraorbital	*Xenomystus*	Measurement [33]	418 HP	100	x/y
12	Neuromast	Free neuromast	*Xenopus*	Measurement [34]	25–40 HP	300	x/y

**Fig 3:**
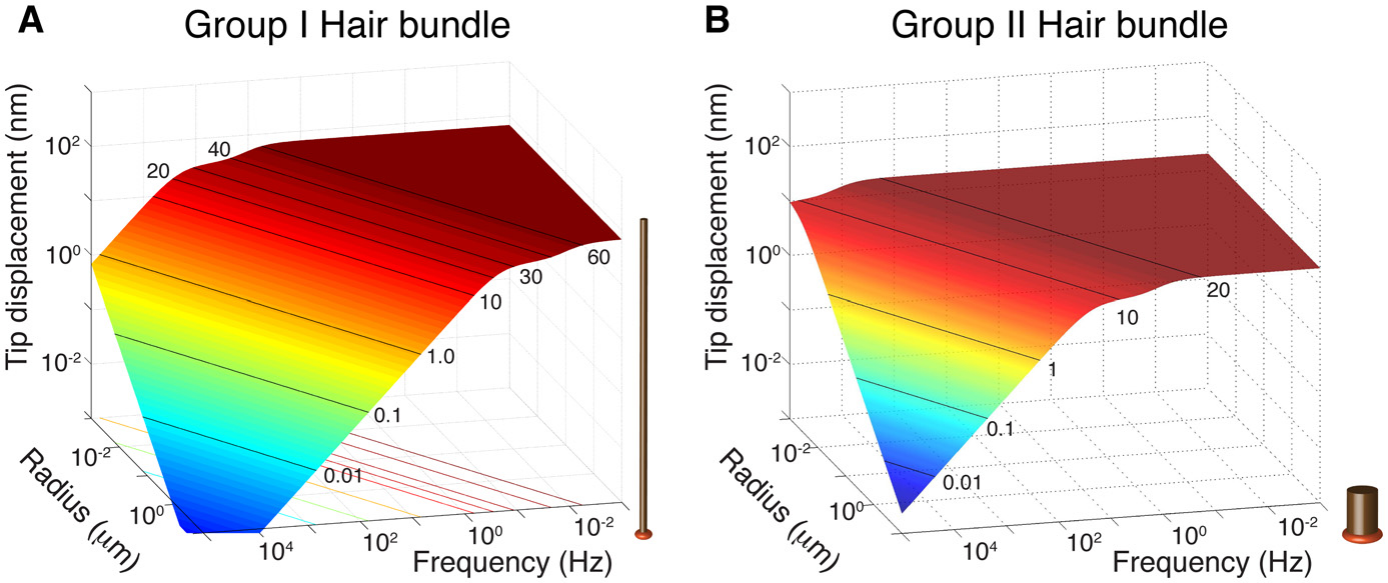
Tip displacement of the hair bundle model due to Brownian motion. Calculated root-mean-square displacement of modelled hair bundle tips as a function of frequency and bundle radius. As input parameters we used (**a**) the low-frequency Group I hair bundle and (**b**) the high-frequency Group II hair bundle (see text). Please note that the frequency axis is inverted for clear display of the roll-off frequencies. The low-frequency plateau is only dependent on the elasticity constant [(Eq 11)]. Hair bundle maximum tip displacement equals 68 nm for Group I in (**a**) and 0.6 nm for Group II in (**b**). The roll-off frequency depends on hydrodynamic friction, hair bundle morphology and endolymph viscosity [(Eq 10)].

Characteristics and source of amplitude spectra in Fig 3 based on published measurements. The first column refers to the numbered lines in Fig 3. The roll-off frequencies in column 6 are indicated as a low-pass (LP), band-pass (BP), or high-pass (HP) filter. The last column indicates whether data were originally presented as acceleration (x/y”), velocity (x/y’) or displacement (x/y) data. The cephalopods curves 3 and 4 in Fig 3, were rescaled according to Fig 7 in [29] and the maximal excursion was taken from *Allotheutis* [30].

### Neural response curves

We constructed frequency sensitivity, i.e. hair bundle displacement plots vs. frequency across a range of vertebrate species and hair cell mechanoreceptor types. To obtain displacement data, we rescaled original data from velocity or acceleration data (For sources see Table 2). If only gain was provided, the absolute excursions were derived from the text of other publications as indicated in Table 2. Each hair cell demonstrates maximum displacement amplitude of the frequency characteristics where output saturation occurs, i.e. a higher amplitude stimulus will not further increase neural output parameters such as spike rate [4]. Based on these data we recognised two distinctly separated groups of hair cells. The first group (Group I) contains hair cells that react to frequency input ranging from 0.004–30 Hz, and the second group (Group II) contains hair cells that react to frequency input ranging from 10 Hz to 150 kHz.

### Hair bundle model parameters

The model input for Group I and II hair bundles is listed in Table 1. For low-frequency hair cells (Group I), the length of the kinocilium is about 70 μm and the radius 0.5–1 μm [17]. This equals a length/radius ratio of 70–140. The longest stereovilli are about 14 μm long. To take the contribution of the stereovilli into account regarding the mechanical properties of the hair bundle, we use a length/radius ratio for the modelled rod of 30–60. The rod is considered as an elongated (i.e. prolate) body to which Stokes’ law for viscous flow is applicable. Conveniently, the force calculated by Stokes’ law for a single sphere of small dimensions is modified for a prolate body [35] by an equivalent radius *r*_*eq*_. With a length/radius ratio of 30 to 60, the equivalent radius *r*_*eq*_ ranges from 10 to 13. The dimension of vestibular hair cell stereovilli as well as the diameter of cochlear inner and outer hair cell stereovilli differs widely amongst metazoans. Our approach—using different equivalent radii—allows for the inclusion of the large observed variation in both kinocilia and stereovilli arrangements and morphologies. For high-frequency hair cells (Group II), the length of the hair bundle rod measures about 7 μm and the radius 3.5 μm (mean values for various receptors), which corresponds to a length/radius ratio of 2. The equivalent radius equals 1 (i.e. spheroid structure [36]).

To our knowledge, no direct stiffness measurements of Group I hair bundles exist. However, we used our hair bundle model to calculate the stiffness of a Group I hair bundle from the magnitude of movement of free hair bundle tips. Brownian Movements of the tips of free ampullary bundles of the glass eel, and of particles in the endolymph next to the hair bundle tips measure 68 nm at a bundle-length of 65 μm [17]. This value has been argued to be surprisingly large [14,17].

With (Eq 11) we calculated the elasticity-constant *κ* to be 44 nN/m using the observed Brownian Motion of 68 nm. Similarly, we calculated Brownian Motion for the Group I hair bundle substituting the measured stiffness of 500 nN/m [5,37] in (Eq 11).

## Results

A hair bundle exhibits a mechanical response to successive bombardments from groups of water molecules, which result in the Brownian Motion. Our results show that hair bundle displacement amplitude is mostly affected by Brownian Motion at lower frequencies, where it is independent of friction and bundle-radius (Fig 3 and Eq 11 in Methods). At higher frequencies (lower left regions in Fig 3a and 3b), the amplitude of displacement decreases with stiffness, friction and bundle radius (Eq 10 in Methods) and therefore the effect of Brownian Motion on the hair bundle tip deflection is much reduced.

**Fig 4:**
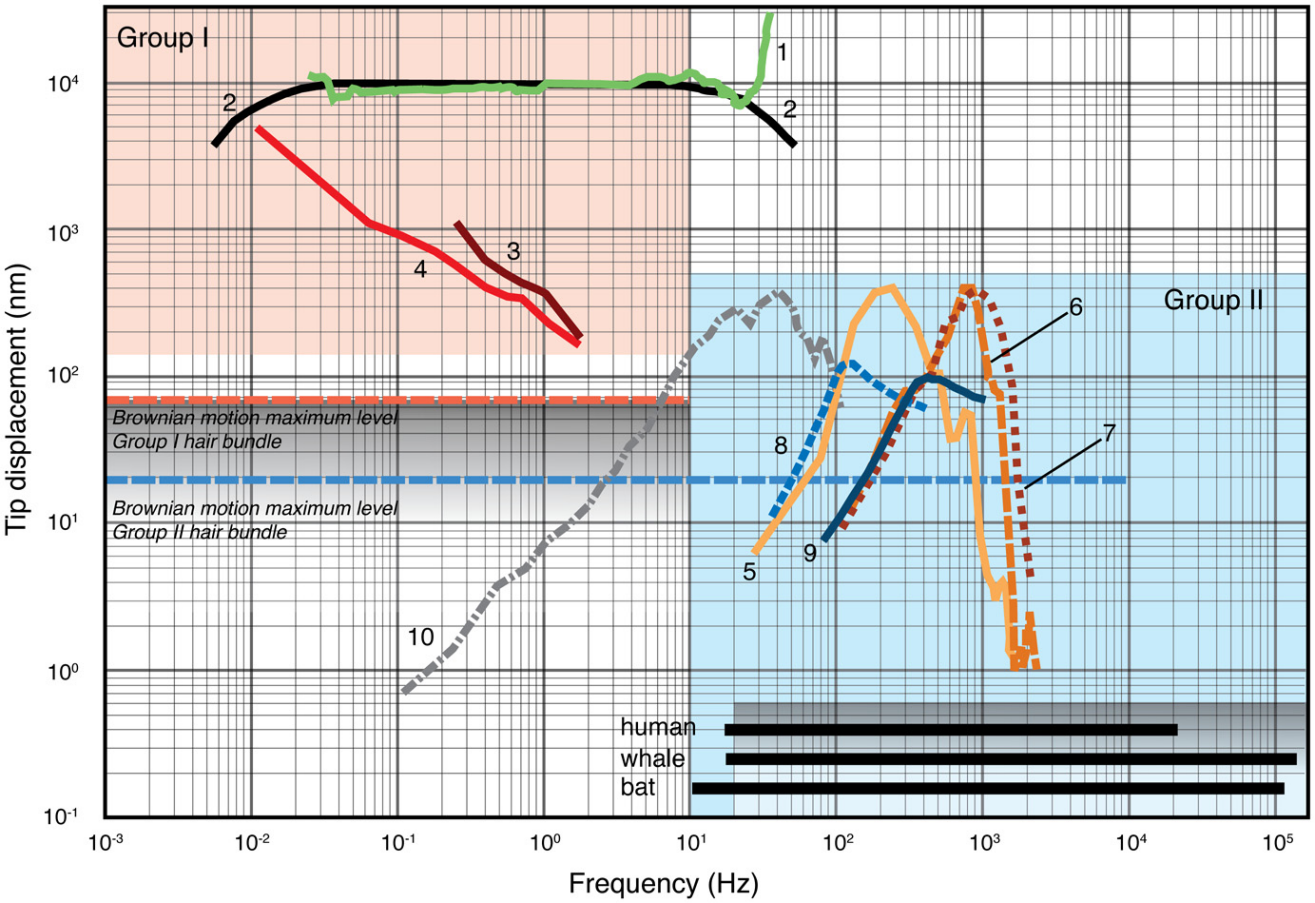
Stimulus displacement and frequency response of hair-cell mechanoreceptors. We compiled and transformed data from a range of vertebrate hair cells into displacement vs. frequency response curves (see Methods and Table 1). Neural output saturation occurs above each curve. This Fig demonstrates the complementary character of Group I hair cells (ampullary-, cephalopod statocyst- and otolith) compared to Group II sensors (lateral line, free neuromasts and cochlea) in frequency and sensitivity as indicated by the red (Group I) and blue (Group II) areas. The graded shaded areas and dashed horizontal lines indicate the Brownian motion low-frequency plateau from our numerical experiments (Fig 1). The bars along the abscissa indicate the frequency-ranges for the auditory system of (h) human (w) whales and (b) bats [39]; 1, ampulla of *Opsanus* [31]; 2, *Homo* [2]; 3,4, statocyst of *Octopus* and *Sepia* [29,30]; 5–7, cochlea of *Cavia* [32]; 8, lateral line of *Acerina* [33]; 9, *Xenomystus* [33]; 10, free neuromast of *Xenopus* [34]. This figure clearly demonstrates that the SNR for both Group I and II hair cells is in the order of magnitude of about 100. With a Brownian Motion displacement amplitude of 100 nm, the SNR for Group II hair cells would be about 1.

Movement of the hair bundle modulates the hair cell’s neural output [6]. To compare hair cell neural responses as a function of hair bundle displacement across vertebrate hair cells, we combined frequency sensitivity data from a wide range of mechanoreceptors and species based on both measurements and models (see Methods). This analysis shows that vertebrate sensory hair cells translate displacement, spanning an impressive amplitude range from 10^−1^ to 10^4^ nm and a frequency range of 10^−3^ to 10^5^ Hz (Fig 4). Furthermore, displacement and frequency response divide hair cells into two distinctly separated groups (Fig 4). The first group (Group I) consists of hair cells that react to frequency input ranging from 0.004–30 Hz. These are those that are found in the semicircular canals, otolith organs [2,27] and cephalopod statocysts [29,30] and require large hair bundle tip-displacements of 300 nm—10 μm. The second group (Group II) consists of hair cells that react to frequency input ranging from 10 Hz to 150 kHz. They are 30–100 times more sensitive with maximal hair bundles tip-displacement of only 100–300 nm. This group includes hair cells of the lateral line system [38], free neuromasts [34] and the cochlea of the auditory system [32]. Although considerable variation in morphology of hair bundles is found, the length of Group I hair bundles is about 70 μm [17] making them, on average, roughly 10 times longer than Group II bundles, which measure around 7 μm [5].

Using our model, we can calculate bundle displacement due to Brownian Motion for generalised Group I and Group II hair bundles. We use input parameters for our model as listed in Table 1 (see also Methods). The rms bundle displacement due to Brownian motion is 70 nm and 20 nm or less for a Group I and II hair bundle respectively (Fig 3 and horizontal dotted lines in Fig 4). With these values we can consider the order of magnitude of the hair bundle displacement signal-to-noise ratio (SNR). For a Group I hair cell, measured displacement is 500 nm—10 μm (line 4 and 1 in Fig 4 respectively) and noise due to the Brownian Motion is maximal 70 nm, resulting in a SNR of about 10–140. For a Group II hair cell, measured displacement is 100–400 nm and noise due to the Brownian Motion is maximal 20 nm, resulting in a SNR of an order of magnitude 5–20.

## Discussion

Our data support the hypothesis that SCC hair cells have adapted hair bundle properties to circumvent signal input masking by Brownian Motion. Our model estimates the observed Brownian motion induced hair bundle displacement for both Group I and II hair bundles, using the physical parameters listed in Table 1. As mentioned in the introduction, the available width of the cupular canals [19] (2–3 μm) should allow the observed 68 nm displacement of free ampullary hairs of the glass eel (*Anguilla* sp.) [17]. For Group II hair bundles, our model predicts a Brownian motion tip displacement of 20 nm or less at the corner frequency. This is in order of magnitude agreement with the measured maximal value of 2 nm [14] that is rapidly reached at higher frequencies. To detect low frequencies <10 Hz, earlier theoretical work [2,3] shows that hair bundles need to reduce stiffness and increase length compared to Group II hair bundles, which is consistent with observations [17]. The stiffness of a cochlear hair bundle measures 500 nN/m [5,37] and we calculated the stiffness of an Group I bundle to be 44 nN/m making the Group I bundles 10 times less stiff than Group II bundles, corroborating previous estimates [40]. Given that Group I hair bundles are about 10 times longer than those in Group II, this length increase (i.e mass increase) and stiffness decrease also increases the deflection of the hair bundle caused by Brownian Motion.

As a consequence of increasing Brownian motion noise, the signal to noise ratio (SNR) for the Group II hair cells decreases about a factor 4. The Group II hair cells SNR decreases from 5–20 to only 1.24–5. This outcome is also supported by predictions based on micromechanical models [10] suggesting that below 20 Hz Brownian Motion noise is about 50 nm above the relevant noise level for auditory hairs (Fig 3 in [10]). For stimuli near the detection threshold, noise resulting from the Brownian Motion can mask the displacement signal [9,10,15] and several passive and active amplification mechanisms have been discovered that should allow cochlear [14,41,42], saccular [16] and SSC [43] hair cells to detect thresholds below Brownian Motion at their peak frequency sensitivity. For example, increasing the displacement amplitude noise by a factor of 1.4 can increase signal detector by stochastic resonance, but at a factor of 10 the signal disappears [14]. Another example is the phenomenon of binaural masking level difference (BMLD) where an identical signal played at each ear, masked by identical noise at each ear, can be made 12–15 dB (i.e. sound pressure level difference factor 4.0–5.6) more detectable by inverting the waveform of either the tone or noise at one ear [44]. However, even with such significant neural amplification mechanisms, the displacement signal still has to be in the order of magnitude of the Brownian Motion noise to be detected. However, at frequencies < 10 Hz, most group II hair bundles (except for the free neuromast of *Xenopus* (line 10 in Fig 4) would be masked by Brownian Motion noise that is more than two order of magnitude larger (Fig 4) and therefore unlikely to be detected by neural amplification mechanisms. Thus, to detect frequencies < 10 Hz we observed that hair bundles have decreased their sensitivity to overcome the masking effects of Brownian Motion. Consequently, large momentum stimuli are required to overcome this decreased sensitivity. In the SCC increasing stimulus momentum can be accomplished by either increased mass or reduced friction [3]. Because fluid friction can only be reduced to a very limited extent, we suggest that the only viable option may be to increase mass of the fluid system stimulating the mechanoreception hair bundle.

We suggest that low frequency rotation detection required the evolution of guiding structures that provide larger momentum stimuli, such as stimulation by a larger mass of endolymph flow, which provide an explanation for the presence of large semicircular canals. Gradual selection for increased mass over evolutionary time will have allowed for lower frequency mechanoreception. In addition, higher frequency components of the stimuli to the hair bundles are filtered out due to inertial effects of increased mass. Therefore, an alternative explanation for the presence of semicircular canals and otoliths may be selection for increased mass to simply isolate low frequency stimuli. However, our data suggest that despite such better-tuned low frequency stimuli, Brownian Motion noise alone under natural conditions would still reduce the SNR for Group II cells and therefore reduce the SNR and mask the embedded input stimulus.

A basic assumption of our models is that the flow coming from the semicircular ducts acts directly on the hair bundles through the space between the epithelium of the crista and the cupula (the subcupular space). This view was put forward for the first time by Dohlman [21] supported by experiments [20] and implemented and tested by hydrodynamical models of the SCC [3]. When the subcupular space is very narrow almost no flow can occur, and therefore the fluid velocity is low. On the other hand for a very wide space, the fluid velocity is also low. Thus in between, a maximal endolymph velocity must occur. Hydrodynamical models based on subcupular space activation of the cupula predicted that the length- and diameter-ratios of the semicircular canal and the subcupular space indeed maximized endolymph velocity, which was confirmed by measurements [3]. These models and experimental data thus support the notion that the subcupular space activates the cupula. Additionally, the cupula is at its distal end firmly anchored to the ampullar roof and also embedded in a mucopolysaccharide-gel [18]. This implies that endolymph forces and Brownian Motion forces are interacting directly at the hair bundle, instead via a relatively massive cupular structure. Interestingly, in the primitive vertebrate *Myxine* no cupula is present [24], which supports the above view. Either way, for our considerations regarding Brownian Motion, it is in our view not relevant whether the endolymph flow and hair bundles motion move the cupula or that the cupula would move the hair bundles. The canaliculi in the cupula are also filled with mucopolysaccharides albeit here much more dilute. This probably keeps the mean distal positions of the kinocilia stable, also during stimulation. But, nevertheless, the entire hair bundles will still undergo the influence of BM forces.

Rüsch and Thurm [17] discovered that mechanical stimuli may induce undulatory movements of the kinocilia. Electrical stimuli had no effect. This lead us to think that the cupula may slide along the (many) kinocilia in a vertical direction. In this way, the subcupular space might be widened, and consequently its amplifying function weakened. In this way, the combined movements of the kinocilia might protect hair bundles for overstimulation (see [3]). Obviously, these speculations require experimental validation.

Our simplified model predicts that for hair bundles the following features are of importance: (1) The low frequency plateau i.e. the amplitude of Brownian motion is only dependent on hair bundle elasticity (*κ*) (Eq 11). (2) The roll-off frequency (i.e. the inverse of the time constant *T*_*c*_ *= γ/κ*) is dependent on elasticity (*κ*), hair bundle radius (*r*) and hair bundle length (expressed in equivalent radius *r*_*eq*_, see text with Eq 1). Therefore, the roll-off frequency increases with bundle stiffness. As the ampullar bundles are highly compliant, their roll-off frequencies are low. From Eqs 1 and 10 and their descriptions, it follows that roll-off frequency is also inversely proportional to hair bundle length, but not to radius. This implies that for our analysis hair bundle width is not relevant. The biological variation of hair bundle lengths found within Group I (vestibular system) and within Group II hair bundles (lateral line, auditory system) is small compared to the difference between these groups. The architecture of crista and cupula varies greatly among all 45,000 species of extant vertebrates. Future work will be required to establish a more elaborate overview of hair cell morphologies, their frequency responses and effects of Brownian Motion.

Molecular data favours the explanation that the insensitive hair cells found in the SCC share a common ancestor with hair cells found in the lateral line system and in free neuromasts [45,46]. Both of these structures contain sensitive and high frequency sensors, suggesting that the insensitive semicircular canal sensors evolved from an ancestral design with high sensitivity. Generally, selective pressure increases sensitivity for sensory organs over evolutionary time [47]; here we hypothesize a sensory system for which selection appears to have favoured a *reduction* in sensitivity. Specifically, our data suggest that by reducing sensitivity, low frequency sensors such as the SCC circumvent Brownian Motion noise overload. This notion is also supported by the discovery of hair cells in the coronal organ of ascidians [48–50]. The coronal organ is found in the inflow-opening of these animals and probably functions to measure the flow of incoming plankton particles. Here, hair cells are found which are remarkably similar in morphology to the type II hair cells of the lateral line and of the auditory system in vertebrates. Thus we speculate that a possible succession in evolution might have occurred from hair cells in the coronal organ of lower chordates (measuring flow), to free neuromasts and lateral line cells in lower vertebrates (measuring flow and sound [51,52]), to auditory cells in the labyrinth (measuring sound) and, finally, to type I cells in the vestibulum of the labyrinth (measuring gravity and rotation).

The vertebrate semicircular canal system plays an important role in weighing multimodal visual and proprioceptory input signals to maintain equilibrium and balance [53,54] and is thus essential in ensuring that an animal’s stance and posture are well-suited to the behaviours that may need to be initiated or are already underway. Successful detection of very low frequency mechanical stimuli allows for coordinated and controlled movements through complex environments, opening previously unexploited niches to early vertebrates.

## References

[1] Spoor F, Garland T, Krovitz G, Ryan TM, Silcox MT, Walker A. (2007) The primate semicircular canal system and locomotion. PNAS 104: 10808–10812. 10.1073/pnas.0704250104 17576932 PMC1892787

[2] Muller M (1999) Size Limitations in Semicircular Duct Systems. Journal of Theoretical Biology 198: 405–437. 10.1006/jtbi.1999.0922 10366494

[3] Muller M (1994) Semicircular Duct Dimensions and Sensitivity of the Vertebrate Vestibular System. Journal of Theoretical Biology 167: 239–256. 10.1006/jtbi.1994.1066

[4] Rabbitt RD, Boyle R, Highstein SM (1995) Mechanical indentation of the vestibular labyrinth and its relationship to head rotation in the toadfish, *Opsanus tau*. Journal of Neurophysiology 73: 2237–2260. 7666136 10.1152/jn.1995.73.6.2237

[5] Hudspeth AJ (1989) How the ear's works work. Nature 341: 397–404. 10.1038/341397a0 2677742

[6] Kung C (2005) A possible unifying principle for mechanosensation. Nature 436: 647–654. 10.1038/nature03896 16079835

[7] Fritzsch B, Straka H (2014) Evolution of vertebrate mechanosensory hair cells and inner ears: toward identifying stimuli that select mutation driven altered morphologies. J Comp Physiol A 200:5–18 10.1007/s00359-013-0865-z PMC3918741 24281353

[8] Denk W, Webb W (1989) Thermal-noise-limited transduction observed in mechanosensory receptors of the inner ear. Phys Rev Lett 63: 207–210. 10.1103/PhysRevLett.63.207 10040807

[9] Denk W, Webb WW (1992) Forward and reverse transduction at the limit of sensitivity studied by correlating electrical and mechanical fluctuations in frog saccular hair cells. Hearing Research 60: 89–102. 1500380 10.1016/0378-5955(92)90062-r

[10] Svrcek-Seiler WA, Gebeshuber IC, Rattay F, Biro TS, Markum H (1998) Micromechanical models for the Brownian motion of hair cell stereocilia. Journal of Theoretical Biology 193: 623–630. 10.1006/jtbi.1998.0729 9745758

[11] Brown R (1828) A brief account of microscopical observations on the particles contained in the pollen of plants and of the general existence of active molecules in organic and inorganic bodies. Edinburgh New Philosophical Journal: 358–371.

[12] Einstein A (1906) Über die von der molekularkinetischen Theorie der Wärme geforderte Bewegung von in ruhenden Flüssigkeiten suspendierten Teilchen. Annalen der Physik F4: 549–560.

[13] Curthoys IS, Markham CH, Curthoys EJ (1977) Semicircular duct and ampulla dimensions in cat, guinea pig and man. J Morphol 151: 17–34. 10.1002/jmor.1051510103 830956

[14] Jaramillo F, Wiesenfeld K (1998) Mechanoelectrical transduction assisted by Brownian motion: a role for noise in the auditory system. Nature Neuroscience 1: 384–388. 10196528 10.1038/1597

[15] Kozlov AS, Andor-Ardó D, Hudspeth AJ (2012) Anomalous Brownian motion discloses viscoelasticity in the ear's mechanoelectrical-transduction apparatus. PNAS 109: 2896–2901. 10.1073/pnas.1121389109 22328158 PMC3286975

[16] Kozlov AS, Baumgart J, Risler T, Versteegh CPC, Hudspeth AJ (2011) Forces between clustered stereocilia minimize friction in the ear on a subnanometre scale. Nature 474: 376–379. 10.1038/nature10073 21602823 PMC3150833

[17] Rüsch A, Thurm U (1990) Spontaneous and electrically induced movements of ampullary kinocilia and stereovilli. Hearing Research 48: 247–263. 2272934 10.1016/0378-5955(90)90065-w

[18] Rüsch A, Thurm U (1989) Cupula displacement, hair bundle deflection, and physiological responses in the transparent semicircular canal of young eel. Pflugers Arch 413: 533–545. 2740206 10.1007/BF00594186

[19] Nagel G, Thurm U (1986) Strukturen der Reizkraft-Ubertragung auf die Sinneshaare (Stereovilli) der Bogengangsampullen niederer Vertebraten. Verh Dtsch Zool Ges: 228.

[20] Suzuki M, Harada Y, Sugata Y (1984) An experimental study on a function of the cupula. Effect of cupula removal on the ampullary nerve action potential. Arch Otorhinolaryngol 241: 75–81. 6335027 10.1007/BF00457920

[21] Dohlman GF (1980) Critical Review of the Concept of Cupula Functon. Acta Oto-Laryngologica 90: 2–30. 6272534

[22] Kondrachuk AV, Shipov AA, Astakhova TG, Boyle RD (2011) Current trends in mathematical simulation of the function of semicircular canals. Hum Physiol 37: 802–809. 10.1134/S0362119711070164

[23] Dohlman GF, Boord RL (1964) The effects of cupular removal on the activity of ampullary structures in the pigeon. Acta Oto-Laryngologica 57: 507–516. 14181098 10.3109/00016486409137113

[24] Jørgensen JM (1998) Structure of the hagfish inner ear In: *The biology of hagfishes*. Springer Netherlands pp. 557–563.

[25] Reif F (1965) Fundamentals of statistical and thermal physics. New York: McGraw-Hill.

[26] Einstein A (1907) Theoretische Bemerkungen Über die Brownsche Bewegung. Z Elektrotech Elektrochem 13: 41–42.

[27] Grant JW, Cotton JR (1990) A model for otolith dynamic response with a viscoelastic gel layer. Journal of vestibular research 1: 139–151. 1670147

[28] Telban RJ, Cardullo FM, Guo L (2000) Investigation of mathematical models of otolith organs of human centered motion cueing algorithms. Proc. AIAA, Modeling and Simulation Technologies.

[29] Williamson R, Budelmann BU (1985) The response of the *Octopus* angular acceleration receptor system to sinusoidal stimulation. Journal of Comparative Physiology A: Sensory, Neural, and Behavioral Physiology 156: 403–412.

[30] Williamson R (1990) The responses of primary and secondary sensory hair cells in the squid statocyst to mechanical stimulation. Journal of Comparative Physiology A: Sensory, Neural, and Behavioral Physiology 167: 655–664.

[31] Highstein SM, Rabbitt RD, Boyle R (1996) Determinants of semicircular canal afferent response dynamics in the toadfish, *Opsanus tau*. Journal of Neurophysiology 75: 575–596. 8714636 10.1152/jn.1996.75.2.575

[32] Khanna SM, Ulfendahl M, Flock A (1989) Mechanical response of the outer hair cell region of the isolated guinea pig cochlea *in vitro* In: Wilson JP, Kemp DT, editors. *Cochlear mechanisms*: *structure*, *functions and models*. New York: Plenum Press p. 616.

[33] Wiersinga-Post JEC, van Netten SM (2000) Temperature dependency of cupular mechanics and hair cell frequency selectivity in the fish canal lateral line organ. Journal of Comparative Physiology A: Sensory, Neural, and Behavioral Physiology 186: 949–956. 11138795 10.1007/s003590000147

[34] Kroese ABA, Zalm JM, Bercken J (1978) Frequency response of the lateral-line organ of *Xenopus laevis*. Pflugers Arch 375: 167–175. 10.1007/BF00584240 567787

[35] Happel J, Brenner H (1983) Low Reynolds Number Hydrodynamics: With Special Applications to Particulate media. Springer

[36] Shatz LF (1998) The effect of shape on the hydrodynamics of a hemispheroid projecting from a plate in irrotational fluid. Phys Fluids 10: 2177–2187. 10.1063/1.869739

[37] Howard J, Hudspeth AJ (1988) Compliance of the hair bundle associated with gating of mechanoelectrical transduction channels in the bullfrog's saccular hair cell. Neuron 1: 189–199. 2483095 10.1016/0896-6273(88)90139-0

[38] van Netten SM, Kroese ABA (1987) Laser interferometric measurements on the dynamic behaviour of the cupula in the fish lateral line. Hearing Research 29: 55–61. 10.1016/0378-5955(87)90205-X 3654397

[39] Sales G, Pye D (1974) Ultrasonic communication by animals. Halsted Press.

[40] Muller M (1990) Relationships between semicircular duct radii with some implications for time constants. Netherlands Journal of Zoology 40: 173–202.

[41] Dallos P (2008) Cochlear amplification, outer hair cells and prestin. Current Opinion in Neurobiology 18: 370–376. 10.1016/j.conb.2008.08.016 18809494 PMC2630119

[42] Dallos P, Wu X, Cheatham MA, Gao J, Zheng J, Anderson CT, et al (2008) Prestin-based outer hair cell motility is necessary for mammalian cochlear amplification. Neuron 58: 333–339. 10.1016/j.neuron.2008.02.028 18466744 PMC2435065

[43] Rabbitt RD, Boyle R, Highstein SM (2010) Mechanical amplification by hair cells in the semicircular canals. PNAS 107: 3864–3869. 10.1073/pnas.0906765107 20133682 PMC2840494

[44] Gilbert HJ, Shackleton TM, Krumbholz K, Palmer AR (2015) The Neural Substrate for Binaural Masking Level Differences in the Auditory Cortex. J Neurosci 35: 209–220. 10.1523/JNEUROSCI.1131-14.2015 25568115 PMC4287143

[45] Collazo A, Fraser SE, Mabee PM (1994) A dual embryonic origin for vertebrate mechanoreceptors. Science 264: 426–430. 8153631 10.1126/science.8153631

[46] Fritzsch B, Pauley S, Beisel KW (2006) Cells, molecules and morphogenesis: the making of the vertebrate ear. Brain Res 1091: 151–171. 10.1016/j.brainres.2006.02.078 16643865 PMC3904743

[47] Manley GA, Popper AN, Fay RR, editors (2004) Evolution of the vertebrate auditory system. Berlin: Springer-Verlag.

[48] Caicci F, Burighel P, Manni L (2007) Hair cells in an ascidian (Tunicata) and their evolution in chordates. Hearing Research 231: 63–72 17611058 10.1016/j.heares.2007.05.007

[49] Burighel P, Caicci F, Zaniolo G, Gasparini F, Degasperi V, Manni L (2008) Does hair cell differentiation predate the vertebrate appearance? Brain Research Bulletin 75: 331–334 10.1016/j.brainresbull.2007.10.012 18331894

[50] Burighel P, Caicci F, Manni L (2011) Hair cells in non-vertebrate models: lower chordates and molluscs. Hearing Research 273: 14–24 10.1016/j.heares.2010.03.087 20430071

[51] Kalmijn AJ (1988) Hydrodynamic and acoustic field detection In: Sensory biology of aquatic animals. (ed: Atemas J, Fay RR, Popper AN, Tavolga WN) New York, Springer pp 83–136.

[52] Kalmijn AJ (1989) Functional evolution of lateral line and inner ear sensory systems In: The mechanosensory lateral line, neurobiology and evolution. (ed:Coombs S, Gorner P, Münz H) New York, Springer pp 187–216

[53] Nashner LM, Black FO, Wall C (1982) Adaptation to altered support and visual conditions during stance: patients with vestibular deficits. J Neurosci 2: 536–544. 6978930 10.1523/JNEUROSCI.02-05-00536.1982 PMC6564270

[54] Allum JH, Honegger F, Schicks H (1994) The influence of a bilateral peripheral vestibular deficit on postural synergies. J Vestib Res 4: 49–70. 8186863

